# Low Physical Activity and High Screen Time Can Increase the Risks of Mental Health Problems and Poor Sleep Quality among Chinese College Students

**DOI:** 10.1371/journal.pone.0119607

**Published:** 2015-03-18

**Authors:** Xiaoyan Wu, Shuman Tao, Yukun Zhang, Shichen Zhang, Fangbiao Tao

**Affiliations:** 1 Department of Maternal, Child & Adolescent Health, School of Public Health, Anhui Medical University, Hefei, China; 2 Anhui Provincial Key Laboratory of Population Health & Aristogenics, Hefei, China; Institute of Automation, Chinese Academy of Sciences, CHINA

## Abstract

**Objective:**

To test the independent and interactive associations of physical activity (PA) and screen time (ST) with self-reported mental health and sleep quality among Chinese college students.

**Method:**

Data were collected in October, 2013. The gender, age, residential background, body mass index (BMI), perceived family economy and perceived study burden were obtained from a total of 4747 college students (41.6% males and 58.4% females). The outcomes were self-reported PA status, ST, anxiety, depression, psychopathological symptoms and sleep quality. Analyses were conducted with logistic regression models.

**Results:**

Overall, 16.3%, 15.9% and 17.3% of the students had psychological problems, such as anxiety, depression and psychopathological symptoms, respectively. The prevalence of poor sleep quality was 9.8%. High ST was significantly positively associated with anxiety (*OR*=1.38, 95%*CI*: 1.15-1.65), depression (*OR*=1.76, 95%*CI*: 1.47-2.09), psychopathological symptoms (*OR*=1.69, 95%*CI*: 1.43-2.01) and poor sleep quality (*OR*=1.32, 95%*CI*: 1.06-1.65). High PA was insignificantly negatively associated with anxiety, depression, psychopathological symptoms and poor sleep. Low PA and high ST were independently and interactively associated with increased risks of mental health problems and poor sleep quality (*p*<0.05 for all).

**Conclusion:**

Interventions are needed to reduce ST and increase PA in the lifestyles of young people. Future research should develop and measure the impacts of interventions and their potential consequences on sleep, health, and well being.

## Introduction

Physical inactivity is the greatest public health problem of the 21st century [1]. Worldwide, 31.1% of adults (15 years or older) are physically inactive. Inactivity rises with age and is increased in high-income countries [2]. The association of physical activity (PA) with mental health has been well established [3–6]. A growing body of literature indicates that PA can have beneficial effects on mental health [7–9] and sleep quality [10] in adolescents and young adults. A previous study has reported a decrease in depression of approximately 8% for each additional hour of exercise undertaken per week [11].

It is well known that economic and technological improvements increase screen time (ST) among young students in developing countries. Meanwhile, evidence for an independent effect of ST on health has been emerging [12]. Increasing amounts of evidence have shown that ST is adversely associated with multiple health problems, such as cardiovascular disease, mental health problems and sleep quality and academic performance in young people [13–14].

Mental health problems presents a growing concern on college campuses [15]. Collectively, there is a growing view that ST and PA are independently associated with mental health among adolescents and the youth [16]. Most recently, these factors have been suggested to operate independently and synergistically to increase risk [17–18]. High ST and low PA levels have been shown to interact to increase psychological problems [19–20]. Physical inactivity and screen-based sedentary behaviors are highly prevalent among young adults, and these habits are likely to continue to later life [21]. The identification of the population group that is at the highest risk of increased ST and physical inactivity enables the improvement of their well being.

The aim of this study was to examine (i) associations of PA and ST with self-reported mental health and sleep quality; and (ii) the interactive effects of PA and ST on mental health and sleep quality among Chinese college students.

## Methods

### Participants

This study was conducted in October 2013 and was approved by the Ethics Committee of Anhui Medical University. Written informed consents was obtained from all of the participants. The participants in this study were drawn from a cross-sectional survey that was conducted at Anhui Medical University, which is located in central China. Participants were recruited by the random cluster method. Six schools were randomly selected, and individuals attending grades 1 through 3 were assessed. All classes in 6 schools and 3 grades were enrolled in this survey. A total of 4915 college students were selected for in this study, resulting in the receipt of 4858 (98.8%) questionnaires, 4747 (97.7%) of which were valid.

### Questionnaire Data

A self-administered questionnaire containing information on sociodemographic indicators, height, weight, PA, ST, mental health and quality of sleep was administered during a 20~30 min session in the classroom. The following socio-demographic characteristics were obtained: age, gender, residential background (urban or rural area), self-reported family economy and self-reported study burden.

PA was assessed with a reliable measure used extensively in the United States as part of the Youth Risk Behavior Survey. The question is as follows: ‘On how many of the past 7 days did you do exercises to strengthen or tone your muscles, such as push-ups, sit-ups, or weight lifting?’ The responses range from 0 to 7 days. High PA was defined as at least three days per week of exercise. The Physical Activity Rank Scale-3 (PARS-3) was used to assess the PA rank of the college students [22]. The PARS-3 is a self-rated questionnaire that assesses PA rank over a 1-month time period. PA rank was measured according to the intensity, time and frequency of exercise, respectively, with the following equation: PA rank = intensity × time × frequency. The resulting score (≤19, 20–42 and ≥43) was used to determine a low, medium or high PA rank, respectively.

The subjects also reported ST using the following question: How many hours per day do you spend on the computer (including playing video or computer games or using a computer for something) and watching TV/video programs on a usual weekday and weekend day, respectively? ‘ The present study categorized ST as ≤2 h/d and >2 h/d.

To fully assess the psychological problems, the mental health of the participants was assessed, including anxiety, depression and psychopathological symptoms. Anxiety was assessed using the self-rating anxiety scale (SAS), which is a standard assessment instrument that has been examined for reliability and validity in the Chinese population [23]. A total standard score of 50 was set as a cut-off value for depression or anxiety. Depression was assessed in the adolescents using the Center for Epidemiologic Studies Depression Scale (CES-D), which is a commonly used freely available self-report measure of depressive symptoms with a 4-factor 20-item structure [24]. All questions have four answer categories as follows: rarely or never (<1 day), some or a little of the time (1–2 days), occasionally or a moderate amount of the time (3–4 days), and most or all of the time (5–7 days). Higher scores on the CES-D indicated greater depressive symptoms. A total standard score of 16 was set as the cut-off value for depression. Psychopathological symptoms were measured using the Multidimensional Sub-health Questionnaire of Adolescents (MSQA) [25], which is a self-reported screening tool to investigate uncomfortable symptoms you actually feel during the last 3 months. It consists of 39 questions on three dimensions: including 17 questions assessing emotional symptoms (e.g., ‘Do you always feel nervous?’), 9 for behavioral symptoms (e.g., ‘Do you always have the impulse to damage something?’) and 13 for social adaptation problems (e.g., ‘Have you always felt unsuited for school life?’). All of these questions have six answer categories, in accordance with the duration of each symptom (none or lasting for < 1 week, lasting for ≥ 1 weeks, lasting for ≥ 2 weeks, lasting for ≥ 1 month, lasting for ≥ 2 months, lasting for ≥ 3 months). For the calculation step, a symptom duration of ≥ 1 week was transformed into a score of 1 (positive items), and a duration of 0 or < 1 week was transformed into a score of 0 (negative items). A score of 1≥8 indicated the presence of psychopathological symptoms. The validity and reliability of the MSQA has been confirmed [26], and the Cronbach alpha (α) coefficient was 0.96.

Sleep problems were measured by the Pittsburgh Sleep Quality Index (PSQI) [27], which is a self-rated questionnaire that assesses sleep quality and disturbances over a 1-month time interval. Nineteen individual items generate seven ‘component’ scores as follows: subjective sleep quality, sleep latency, sleep duration, habitual sleep efficiency, sleep disturbances, the use of sleep medication, and daytime dysfunction. The sum of the scores of these seven components yields one global score, the PSQI scores, which range from 0 to 21. Higher scores indicate worse sleep quality, and a score of more than 7 indicates poor sleep quality, as has been demonstrated in the Chinese population [28].

### Statistical Analysis

Statistical analysis was performed using SPSS version 13.0 (Statistical Package for the Social Sciences). The chi-square and t tests were performed to assess differences in the characteristics between males and females for the categorical and continuous variables, respectively. The logistic regression model was performed to explore the independent and interactive relationship between PA, ST and various psychological problems and poor sleep quality. Odds ratios (ORs) and 95% confidence intervals (95% CIs) were calculated for the explanatory factors and adjusted for confounding factors, including gender, age, residential background, BMI, perceived family economy and perceived study burden. A P < 0.05 was considered to be statistically significant.

## Results

Of the 4747 participants, 41.6% were males with a mean age of 19.26 years (SD = 1.40), and 58.4% were females with a mean age of 19.22 years (SD = 1.41). Overall, 16.3%, 15.9% and 17.3% of the students had anxiety, depression and psychopathological symptoms, respectively. The prevalence of poor sleep quality was 9.8%. A majority of the students reported a low PA level. Compared to the females, the males had a significantly higher age, BMI, higher prevalences of a rural residential background and perceived study burden and psychopathological symptoms. Nevertheless, compared to the males, the females had significantly higher prevalences of low PA and poor sleep quality (Table 1).

**Table 1:** Characteristics of the sample among Chinese college students.

	Total	Male(n = 1973)	Female(n = 2774)	*p* value
Age	19.24±1.41	19.26±1.40	19.22±1.41	0.000
BMI	20.35±2.54	21.18±2.91	19.76±2.04	0.000
Residential background				0.262
Rural	2680(56.5)	1095(55.5)	1585(57.1)	
Urban	2067(43.5)	878(44.5)	1189(42.9)	
Perceived family economy				0.089
Low	1490(31.4)	651(33.0)	839(30.2)	
Medium	3032(63.9)	1237(62.7)	1795(64.7)	
High	225(4.7)	85(4.3)	140(5.0)	
Perceived study burden				0.000
Low	2238(47.1)	874(44.3)	1364(49.2)	
Medium	2458(51.8)	1063(53.9)	1395(50.3)	
High	51(1.1)	36(1.8)	15(0.5)	
ST				0.387
≤2h/day	3758(79.2)	1550(78.6)	2208(79.6)	
>2h/day	989(20.8)	423(21.4)	566(20.4)	
PA				0.000
Low	4381(92.3)	1688(85.6)	2693(97.1)	
High	366(7.7)	285(14.4)	81(2.9)	
PA rank				0.000
Low	3266(68.8)	1123(56.9)	2143(77.3)	
Medium	966(20.3)	466(23.6)	500(18.0)	
High	515(10.8)	384(19.5)	131(4.7)	
Anxiety	772(16.3)	344(17.4)	428(15.4)	0.065
Depression	754(15.9)	320(16.2)	434(15.6)	0.594
Psychopathological symptoms	823(17.3)	373(18.9)	450(16.2)	0.016
Poor sleep	464(9.8)	181(9.2)	283(10.2)	0.240

Values are presented as mean±SD or number(percentage) when appropriate.

BMI: body mass index. ST: screen time. PA: physical activity.

As shown in Table 2, high ST was significant positively correlated with anxiety, depression, psychopathological symptoms and poor sleep quality, and these correlations were maintained after adjusting for gender, age, residential background, BMI, perceived family economy and perceived study burden. High PA was negatively associated with anxiety, depression, psychopathological symptoms and poor sleep quality, but these associations were with not statistically significance. There were progressive increases in the protective effects against depression, psychopathological symptoms and poor sleep with increasing PA rank, which were maintained after adjusting for gender, age, residential background, BMI, perceived family economy and perceived study burden.

**Table 2:** Associations of screen time, physical activity and mental health, sleep among Chinese college students.

	Anxiety			Depressive			Psychopathological symptoms			Poor sleep		
	n (%)	Crude OR	Adjusted OR	n (%)	Crude OR	Adjusted ^a^OR	n (%)	Crude OR	Adjusted OR	n (%)	Crude OR	Adjusted OR
		(95% CI)	(95% CI)		(95% CI)	(95% CI)		(95% CI)	(95% CI)		(95% CI)	(95% CI)
Screen time												
≤2h/day	575(15.3)	Ref.	Ref.	532(14.2)	Ref.	Ref.	587(15.6)	Ref.	Ref.	347(9.2)	Ref.	Ref.
>2h/day	197(19.9)	1.38(1.15–1.65) ^b^	1.49(1.24–1.79) ^b^	222(22.4)	1.76(1.47–2.09) ^b^	1.86(1.55–2.22) ^b^	236(23.9)	1.69(1.43–2.01) ^b^	1.76(1.48–2.10) ^b^	117(11.8)	1.32(1.06–1.65) ^c^	1.40(1.12–1.75) ^b^
PA												
Low	717(16.4)	Ref.	Ref.	707(16.1)	Ref.	Ref.	772(17.6)	Ref.	Ref.	438(10.0)	Ref.	Ref.
High	55(15.0)	0.90(0.67–1.22)	0.86(0.63–1.16)	47(12.8)	0.77(0.56–1.05)	0.74(0.54–1.03)	51(13.9)	0.76(0.56–1.03)	0.68(0.50–0.94) ^c^	26(7.1)	0.69(0.46–1.04)	0.71(0.47–1.08)
PA rank												
Low	566(17.3)	Ref.	Ref.	554(17.0)	Ref.	Ref.	589(18.0)	Ref.	Ref.	338(10.0)	Ref.	Ref.
Medium	130(13.5)	0.74(0.60–0.91)^c^	0.76(0.61–0.93) ^c^	140(14.5)	0.83(0.68–1.02)	0.81(0.66–0.99) ^c^	158(16.4)	0.89(0.73–1.08)	0.82(0.67–0.99) ^c^	89(9.2)	0.88(0.69–1.12)	0.88(0.68–1.13)
High	76(14.8)	0.83(0.64–1.07)	0.78(0.59–1.02)	60(11.7)	0.65(0.49–0.86) ^c^	0.62(0.46–0.83) ^c^	76(14.8)	0.79(0.61–1.02)	0.70(0.53–0.92) ^c^	37(7.2)	0.67(0.47–0.95) ^c^	0.69(0.48–0.99) ^c^

^a^ Adjusted for gender, age, residential background, BMI, perceived family economy and perceived study burden.

^b^ *p*<0.001.

^c^ *p*<0.05 compared with referent.

The interaction effects of PA and ST with the various psychological problems and poor sleep quality are shown in Table 3. We found a significant additive interaction between ST and PA (P<0.05). The risks of anxiety (OR = 0.71, 95%CI: 0.59–0.85), depression (OR = 0.55, 95%CI: 0.46–0.66), psychopathological symptoms (OR = 0.59, 95%CI: 0.50–0.70) and poor sleep quality (OR = 0.78, 95%CI: 0.62–0.98) were significantly lower (P<0.05 for all) in those with low PA and low ST compared with those with low PA and high ST. Further, these associations were maintained in the adjusted models. In particular, the participants with high PA and low ST had the lowest risks of psychopathological symptoms (OR = 0.46, 95%CI: 0.32–0.67) and poor sleep quality compared with the other groups (OR = 0.50, 95%CI: 0.30–0.82).

**Table 3:** Odds ratio (95% CI) associated with the interaction of screen time and physical activity on mental health and sleep quality among Chinese college students.

	Screen time	Low PA			High PA		
		n (%)	Crude OR (95% CI)	Adjusted OR^a^ (95% CI)	n (%)	Crude OR (95% CI)	Adjusted OR^a^ (95% CI)
Anxiety	>2h/day	188 (20.3)	Ref.	Ref.	9(14.3)	0.65 (0.32–1.35)	0.66 (0.31–1.37)
	≤2h/day	529 (15.3)	0.71 (0.59–0.85) ^b^	0.66 (0.55–0.79) ^b^	46(15.2)	0.70 (0.49–1.00)	0.61 (0.43–0.88) ^c^
Depressive	>2h/day	214 (23.1)	Ref.	Ref.	8 (12.7)	0.48 (0.23–1.03)	0.48 (0.22–1.03)
	≤2h/day	493 (14.3)	0.55 (0.46–0.66) ^b^	0.53 (0.44–0.63) ^b^	39 (12.9)	0.49 (0.34–0.71) ^b^	0.45 (0.31–0.66) ^b^
Psychopathological symptoms	>2h/day	224 (24.2)	Ref.	Ref.	12 (19.0)	0.74 (0.39–1.41)	0.66 (0.34–1.29)
	≤2h/day	548 (15.9)	0.59 (0.50–0.70) ^b^	0.60 (0.48–0.68) ^b^	39 (12.9)	0.46 (0.32–0.67) ^b^	0.41 (0.28–0.59) ^b^
Poor sleep	>2h/day	110 (11.9)	Ref.	Ref.	7 (11.1)	0.93 (0.41–2.01)	0.99 (0.43–2.25)
	≤2h/day	328 (9.5)	0.78 (0.62–0.98) ^c^	0.74 (0.58–0.93) ^c^	19 (6.3)	0.50 (0.30–0.82) ^c^	0.48 (0.29–0.81) ^c^

^a^ Adjusted for gender, age, residential background, BMI, perceived family economy and perceived study burden.

^b^ *p*<0.001.

^c^ *p*<0.05 compared with referent.

## Discussion

The findings of this study suggest that high ST is significant positively correlated with anxiety, depression, psychopathological symptoms and poor sleep quality. Meanwhile, low PA and high ST are associated with a reduced prevalence of mental health problems and poor sleep quality.

Increasing evidence is suggesting that PA is associated with numerous health benefits [29–30]. In general, the association of PA with mental health in young people is evident [31]. It has also been demonstrated that PA can influence the mental health of college students[32–33]. Although our study found an insignificant relationship between high PA and mental health, PA rank was found to be significantly correlated with anxiety and psychopathological symptoms. As predicted, an increased PA rank was associated with significant reductions in depression, anxiety, psychological distress, and poor sleep among college students. An undergraduate student sample [34] has been reported with significant differences between the low, medium and high exercise groups in the mental health status, which is in agreement with our research. Moderate PA is important for youth, whose brains are highly plastic [35]. Possible mechanisms include an increase in serotonin or other neurotransmitters associated with the ‘endorphin’ effect in alleviating negative effect. Moreover, PA may influence brain health and cognition in young adults [36–37].

Screen-based technologies are ubiquitous among modern youth and are frequently used, and the consequences of excessive ST on general health, PA, and cognitive and social development have been proven in adolescents and youth [38–39]. A previous study has demonstrated an independent association of ST with psychological health in adults [40]. Recently, associations between reduced psychological wellbeing, lower PA levels and increased ST in adolescents have been reported [11, 17, 20]. Our results suggest that high ST and low PA increase the risk of psychological problems independently and also synergistically in college students. Possible underlying mechanisms for the adverse health effects are complex. One of the ways that ST has been hypothesized to influence health is by displacing time that could otherwise have been used for PA [41]. Furthermore, ST is highly correlated with increased metabolic risk [42], and metabolic risk is associated with poor mental health [43]. This finding may represent a potential mechanism explaining the link between ST and mental health.

Our study also provides interesting information with regard to sleep quality among college students in China. Poor sleep is highly prevalent among college students [44], and is associated with poor mental health [45]. An adverse association between ST and sleep has been shown in a previous study [46]. Moreover, ST has been hypothesized to be a cause of insufficient and low-quality sleep [47], which may be explained by time displacement. With more time spent in front of screens, the youth have less time available to sleep. PA is an efficient remedy and preventative measure for poor sleep [48]. Sleep and PA influence each other through complex, bilateral interactions that involve multiple physiological and psychological pathways [49]. Furthermore, given that PA is associated with reduced symptoms of depression, anxiety and psychological well being, its positive influence on sleep may be mediated by general psychological functioning [48]. The mechanism underlying this relationship requires further investigation. Despite these uncertainties, the evidence is sufficient to endorse continued efforts to limit ST and increase PA for the benefit of sleep quality in this population.

There are some limitations to the present study. First, the cross-sectional design limits the power with which the causal relationships can be determined. Longitudinal studies are needed to understand the causal relationships of PA and ST with mental health and sleep quality. Another limitation that may have affected our results is the fact that the PA and ST levels were assessed by self-reported questionnaires; thus, recall and reporting biases could not be avoided. Third, the question about PA did not specify the type and intensity. Lastly, our study assessed anxiety, depression and symptoms but not clinically diagnosed depression; therefore, different associations may exist when diagnosed mental health is considered as the variable of interest.

Despite the above limitations, the present study highlights the importance of associations of PA and ST with mental health and sleep in Chinese college students. Our results suggest that high ST is significant positively associated with anxiety, depression, psychopathological symptoms and poor sleep quality. Meanwhile, low PA and high ST are associated with increased risks of mental health problems and poor sleep quality. Our findings may suggest that interventions are needed to reduce ST and increase PA in the lifestyles of young people. Future research should develop and measure the impacts of interventions and their potential consequences on sleep, health, and well being.
